# Hereditary papillary renal cell carcinoma primarily diagnosed in a cervical lymph node: a case report of a 30-year-old woman with multiple metastases

**DOI:** 10.1186/1471-2490-13-3

**Published:** 2013-01-15

**Authors:** Carl Ludwig Behnes, Christina Schlegel, Moneef Shoukier, Isabella Magiera, Frank Henschke, Alexander Schwarz, Felix Bremmer, Hagen Loertzer

**Affiliations:** 1 Department of Pathology, University of Göttingen, Robert-Koch-Str. 40, 37075, Göttingen, Germany; 2 Department of Urology, University of Göttingen, Robert-Koch-Str. 40, 37075, Göttingen, Germany; 3 Insitute of Human Genetics, University of Göttingen, Robert-Koch-Str. 40, 37075, Göttingen, Germany; 4 Department of Human Genetics at MVZ Dortmund, Dr. A. Eberhard & Partners, Dortmund, Germany; 5 Institute of Pathology, Reumonstr.28, 33102, Paderborn, Germany; 6 Department of Diagnostic Radiology, University of Göttingen, Robert-Koch-Str. 40, 37075, Göttingen, Germany

**Keywords:** HLRCC, Fumarate hydratase (fh), Papillary renal cell cancer, Leiomyomatosis

## Abstract

**Background:**

Papillary renal cell carcinoma is a rare cancer. Some cases can be attributed to individuals with hereditary renal cell carcinomas usually consisting of the clear cell subtype. In addition, two syndromes with hereditary papillary renal cell carcinoma have been described. One is the hereditary leiomyomatosis and renal cell carcinoma, which is characterized by cutaneous and uterine leiomyomas and renal cell carcinoma mostly consisting of the papillary renal cell carcinoma type II with a worse prognosis.

**Case presentation:**

We describe a case of a 30-year-old woman with hereditary leiomyomatosis and renal cell carcinoma syndrome with extensively metastasized papillary renal cell carcinoma, primarily diagnosed in a cervical lymph node lacking leiomyomas at any site.

**Conclusion:**

Papillary renal cell carcinoma in young patients should be further investigated for a hereditary variant like the hereditary leiomyomatosis and renal cell carcinoma even if leiomyomas could not be detected. A detailed histological examination and search for mutations is essential for the survival of patients and relatives.

## Background

Renal cell carcinoma (RCC) is a rather rare cancer with an incidence of about 71,000 newly diagnosed cases per year in Europe. Approximately 31,000 of these patients die because of RCC [1]. Only a few cases represent hereditary RCCs [2], of which the Von-Hipple-Lindau syndrome (VHL; MIM# 193300) is best known [3]. Furthermore, Birt-Hogg-Dubé syndrome (BHD; MIM# 135150) [4] and tuberous sclerosis (TS1; MIM# 191100 / TS2; MIM# 613254) [5] have been reported to be associated with hereditary RCC mostly of the clear cell or chromophobe subtype. In addition two syndromes associated with hereditary papillary RCC have been described. One is represented by the hereditary papillary renal carcinoma (HPRC; MIM**#** 605074) usually consisting of papillary RCC type I with a better prognosis [6] showing activating mutations in the *met* proto-oncogene (*MET*) on chromosome 7q32 are responsible for HPRC [7]. The other syndrome compromises the hereditary leiomyomatosis and renal cell carcinoma (HLRCC; MIM# 150800), which is characterized by papillary RCC type II with a worse prognosis as well as typically cutaneous and uterine leiomyomas [8]. The HLRCC is caused by heterozygous germline mutations of the fumarate hydratase (*FH*) gene on chromosome 1q34 [9]. The intracellular hypoxia inducible factor (HIF) is stabilized due to an accumulation of FH based on the mutations in this gene region and is responsible for a pseudohypoxic state causing the release of different tumor promoting factors like VEGF, PDGF, and TGF-α [10]. Until now, 125 mutations in the *FH* gene have been described in the Human Gene Mutation Database Professional (HGMD release 2011.4; http://www.biobase.de/hgmd/pro/start.php).

Here we report on a 30-year-old woman suffering on HLRCC initial by presenting with cervical lymph node metastasis and lacking cutaneous or uterine leiomyomas. This case demonstrates the diagnostic challenge of HLRCC especially in case of missing cutaneous leiomyomas.

## Case presentation

### Clinical findings

**Figure 1:**
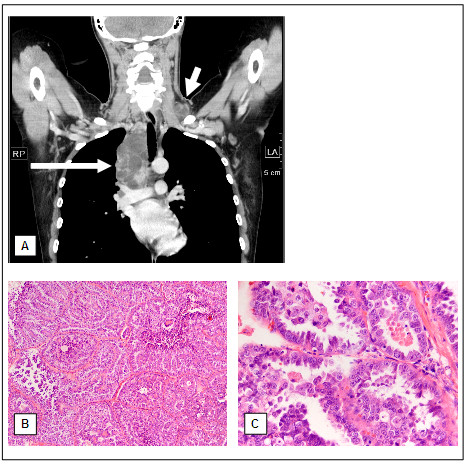
**Coronal reformatted computed tomography image of the initial contrast enhanced chest scan shows rim-enhancing enlarged lymphnodes left supraclavicular between the sternocleidomastoid and the scalene muscles (A, short arrow) and furthermore conglomerate-like tumors in the right upper mediastinum (A, long arrow).** Histologically the lymph node shows infiltrations of a papillary adenocarcinoma (**B**: H&E, x100 / **C**: H&E, x400).

**Figure 2:**
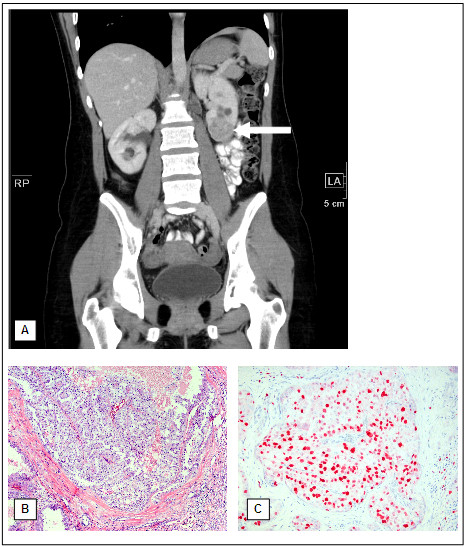
**Coronal reformatted computed tomography image of the initial contrast enhanced abdominal scan shows a renal mass located in the lower pole of the left kidney, appearing inhomogeneous, predominantly less enhancing compared to the normal renal parenchyma **(**A**)**.** Histologically and immunohistochemically examinations of the partial nephrectomy show a tumor similar to the lymph node infiltration with an increased proliferation (**B**: H&E, x100 / **C**: Ki67 immunostaining, x100).

**Figure 3:**
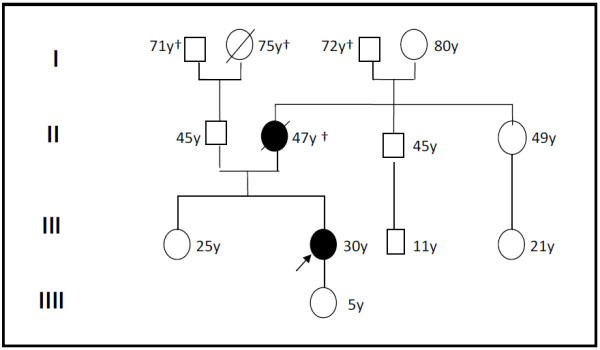
The family pedigree with the described patient (denoted by black arrow) and her mother, who also died of renal cell carcinoma.

A 30-year-old woman was admitted to the hospital because of painless lymphadenopathy in the left neck. Computed tomography of neck and chest revealed multiple lesions up to 9.5 cm in diameter in the left supraclavicular region as well as in the mediastinum (Figure 1A). Based on a supraclavicular lymph node biopsy the diagnosis of a papillary adenocarcinoma was confirmed (Figure 1B and C). In the following abdominal sonography and contrast-enhanced abdominal computertomography identified a 4 cm in diameter inhomogeneous mass in the lower pole of the left kidney (Figure 2A) as well as numerous enlarged paraaortal lymph nodes. Partial nephrectomy of the left kidney revealed a tumor of 4 cm in diameter with a tan to gray cut surface. Paraaortal lymph nodes showed tumor infiltrations. Two weeks later a new hypodense lesion 2.2 cm in diameter was detected in the liver. A clinical check-up after 2 month demonstrated by thoracal and abdominal computertomography numerous suspicious mediastinal and paraaortal lymph nodes (Figure 1A and 2A) and further suspect lesions in the liver. The patient confirmed that her mother died from renal cell carcinoma at the age of 47. Medical records of the mother were not available. No further cases of RCC have been reported in the patient's family (Figure 3).

### Pathological and genetic findings

On macroscopical examination cross sections of cervical lymph node demonstrated a grossly white, partly cystic, and necrotic mass.

Microscopical examination of the cervical lymph nodes revealed a tumor consisting of papillary formations crossed by fibrotic areas. The papillae showed a monolayer of tumor cells with large nuclei and numerous nucleoli as well as atypical mitotic figures. The tumor cells displayed abundant eosinophilic cytoplasm (Figure 1B and C). The immunhistochemical examinations confirmed a partial expression of cytokeratin 7- and RCC-antibodies. Vimentin, especially expressed in clear cell RCC, could only be demonstrated in the papillary stroma but not in tumor cells. A potential metastasis of a thyroid carcinoma could be excluded by the lacking expression of TTF-1 and thyreoglobulin. Based on the also lacking expression of estrogen- and progesteron-receptor a metastasis of an ovarian cancer was assumed to be unlikely. The tumor demonstrates no further specific antibody reaction. Thus, the lymph node metastasis seemed to be most likely caused by papillary RCC.

Macroscopical examination of the partial nephrectomy of the left kidney showed a tumor of 4 cm in diameter with a gray-tan and hemorrhagic cut surface. The tumor did not demonstrate a clear border to the surrounding normal kidney.

Microscopical examination of the partial nephrectomy revealed a papillary RCC type II with the same morphology as in the lymph node. Furthermore lymphangiosis and dissociated tumor islets could be detected. The immunohistochemical results of the renal tumor tissue were identical to the findings of the lymph node. In addition the tumor showed a proliferation rate of 80% by Ki67 staining (Figure 2B and C).

**Figure 4:**
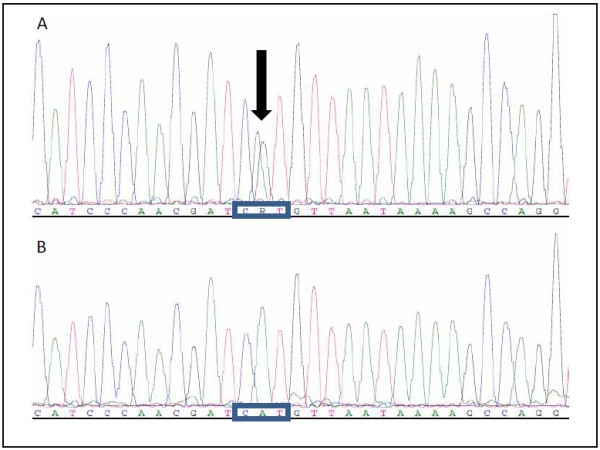
**Chromatograms of mutation analysis in *FH *exon. A**: Heterozygous mutation (black arrow) c.539A>G (CAT→CGT), p.His180Arg in exon 4 of the FH-gene. **B**: Wild type sequence

Molecular genetic analysis did not reveal any mutation in the *met*-oncogene. The subsequent analysis of the *FH* gene revealed a missense mutation (c.539A > G) leading to amino acid substitution H180R at the protein level (Figure 4). This sequence variant has been classified as a pathologic mutation. Furthermore, this mutation could be shown to be associated with a reduced activity of *FH* by approximately 45% [11].

## Conclusion

The papillary RCC is a rare tumor entity normally occuring beyond the age of 60 years being associated with a better prognosis than the more often clear cell RCC [12]. Especially appearing during early lifespan papillary RCC should be tested for an hereditary variant. One of these variants is the HLRCC syndrome associated with different mutations in the *FH* gene [13], which functions as a tumor suppressor gene. Up to now about 180 families with HLRCC have been found worldwide. Some of these mutations are sporadic [14]. In a North American series of male and female patients papillary RCC in HLRCC occurred at a median age of 42 years of patents, in which the youngest patient was 11 years old [15,16].The HLRCC syndrome is characterized by leiomyomas especially of the skin and papillary RCC [8]. In HLRCC patients, leiomyomas of the skin can be observed by the age of 35 years in all affected men and in about 55% of affected woman [17,18]; leiomyomas of the uterus occur in approximately 85% of females [19]. In addition *FH* mutations have also been described in case of uterine fibroids [20]. The papillary RCC in HLRCC shows a penetrance of about 20-25% and mostly consists of a papillary RCC type II with a worse prognosis [12]; most patients die within five years [21]. Because RCC even in HLRCC typically arise unilateral, an immediately nephrectomy is the most efficient therapy [22].

Generally the diagnosis of HLRCC is made by the frequently occurring cutaneous leiomyomas, which can be painful. The patient described in this study showed no cutaneous leimyomas at all and lack leiomyomas of the uterus. Instead of that the patient primarily presented metastases of a papillary RCC in cervical lymph nodes. In the literature one case of an 18-year-old woman from a Dutch family with HLRCC presenting metastases of a RCC in cervical lymph nodes can be found [23]. Because of the here described patient young age the diagnosis was suspicious for a hereditary syndrome such as HPRC or HLRCC. The genetic examination showed a mutation in the *FH* gene typical for HLRCC.

To our best knowledge, a case of HLRCC with a comparable lymph node and distant dissemination without any leiomyomas has not been described before. This case shows the importance of a complete pathological examination including subtyping of papillary RCC and genetic examination also in the absence of cutaneous or uterine leiomyomas.

### Consent

Written informed consent was obtained from the patient for publication of this case report and any accompanying images. A copy of the written consent is available for review by the Editor-in-Chief of this journal.

## Competing interests

The authors declare that they have no competing interests.

## Authors' contributions

CLB and CS constructed the manuscript and carried out pathological examination. CS, HL, and AS were responsible for the clinical and radiological data. HF participated in pathological investigations. SM and IM carried out the genetic examinations. FB was responsible for critical revision of the manuscript and has been involved in drafting it. All authors read and approved the final manuscript.

## Pre-publication history

The pre-publication history for this paper can be accessed here:

http://www.biomedcentral.com/1471-2490/13/3/prepub
